# Drug-eluting balloon to treat immediate post-coronary artery bypass grafting ST-elevation myocardial infarction: a case report

**DOI:** 10.1093/ehjcr/ytae245

**Published:** 2024-05-15

**Authors:** Muhammad Usman Shah, Muhammad Anis Haider, Krishna Poudyal, Mahmoud Loubani, Syed Yaseen Naqvi

**Affiliations:** Joseph Banks Laboratories, University of Lincoln, Beevor St, Lincoln LN6 7DL, UK; Department of Cardiology and Cardiac Surgery, Hull University Teaching Hospitals, Cottingham, UK; Department of Cardiology and Cardiac Surgery, Hull University Teaching Hospitals, Cottingham, UK; Department of Cardiology and Cardiac Surgery, Hull University Teaching Hospitals, Cottingham, UK; Department of Cardiology and Cardiac Surgery, Hull University Teaching Hospitals, Cottingham, UK; Department of Cardiology and Cardiac Surgery, Hull University Teaching Hospitals, Cottingham, UK

**Keywords:** Coronary artery bypass graft, Drug-eluting balloon, Drug-eluting stents, Case report, ST-elevation myocardial infarction

## Abstract

**Background:**

Coronary artery bypass graft (CABG) surgery represents a major cardiovascular operation and may be associated with post-operative ST-elevation myocardial infarction (STEMI) due to graft failure. This is challenging to diagnose and treat as the implanted grafts may be prone to complications when treated percutaneously with drug-eluting stents.

**Case summary:**

A man in his 60 s underwent CABG and developed new persistent ST elevations of 2 mm in anterior leads with no significant chest pain, although, administered with intravenous opiates post-operatively. Transthoracic echocardiography was non-diagnostic. Invasive angiography performed emergently showed a thrombotic occlusion of the mid-left anterior descending artery at the site of the anastomosis with the left internal mammary artery (LIMA) graft. Intervention via the graft was considered high risk of complications, therefore, native coronary arteries were used to approach the occlusion, which was successfully cleared with a combination balloon angioplasty with a semi-compliant and then a drug-eluting balloon. The LIMA started working again with the resolution of ST elevation and no immediate complications.

**Discussion:**

Early post-operative ST elevations in continuous leads should not be ignored as they often may be the only feature of new-onset STEMI. Drug-eluting balloons represent a feasible and possibly safer option than drug-eluting stents to treat these conditions.

**Learning points**

- Diagnosis of ST-elevation myocardial infarction immediately post-coronary artery bypass surgery is challenging, and a high index of suspicion is required for timely diagnosis.
- Angioplasty with drug-eluting balloons represents a safe option to treat anastomotic site thrombotic occlusions in patients with freshly implanted arterial grafts.

## Introduction

Coronary artery bypass graft (CABG) represents a major cardiovascular operation and may be associated with peri-procedural myocardial infarction.^1^ Diagnosis may be challenging^2^ and percutaneous management associated with complications.^3^ We present the case of a middle-aged man who had immediate post-CABG ST-elevation myocardial infarction (STEMI), successfully managed with a drug-eluting balloon (DEB).

## Summary figure

**Figure:**
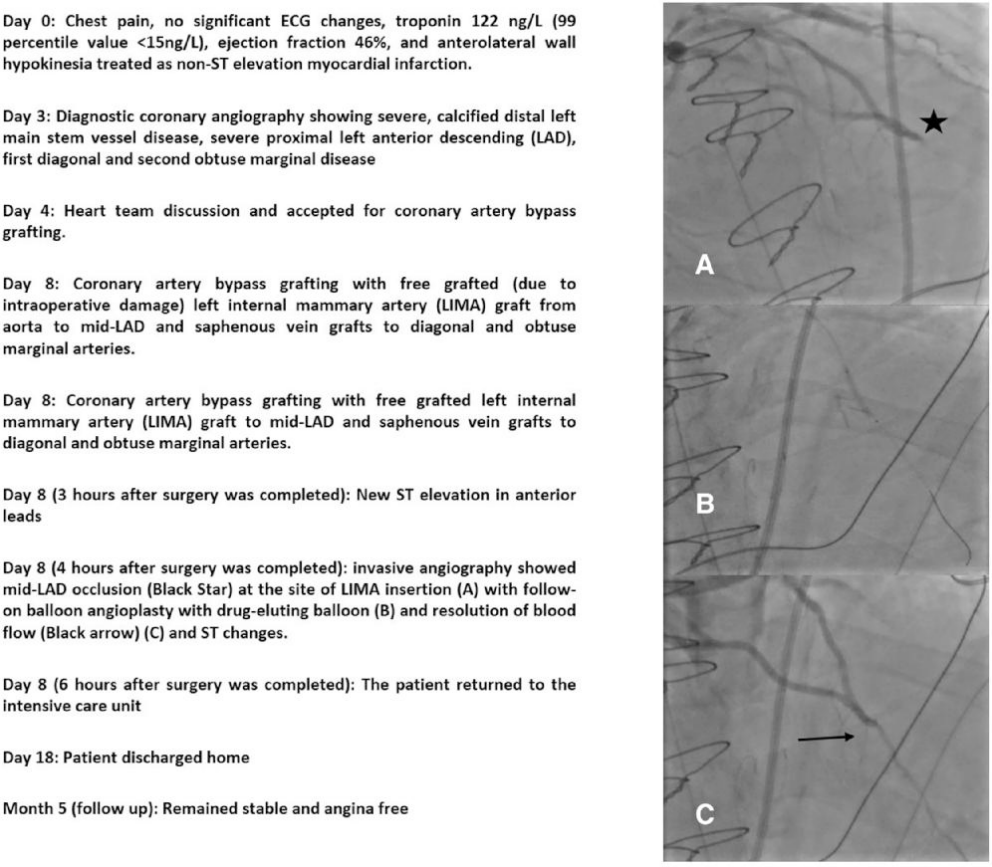


## Case presentation

**Figure 1:**
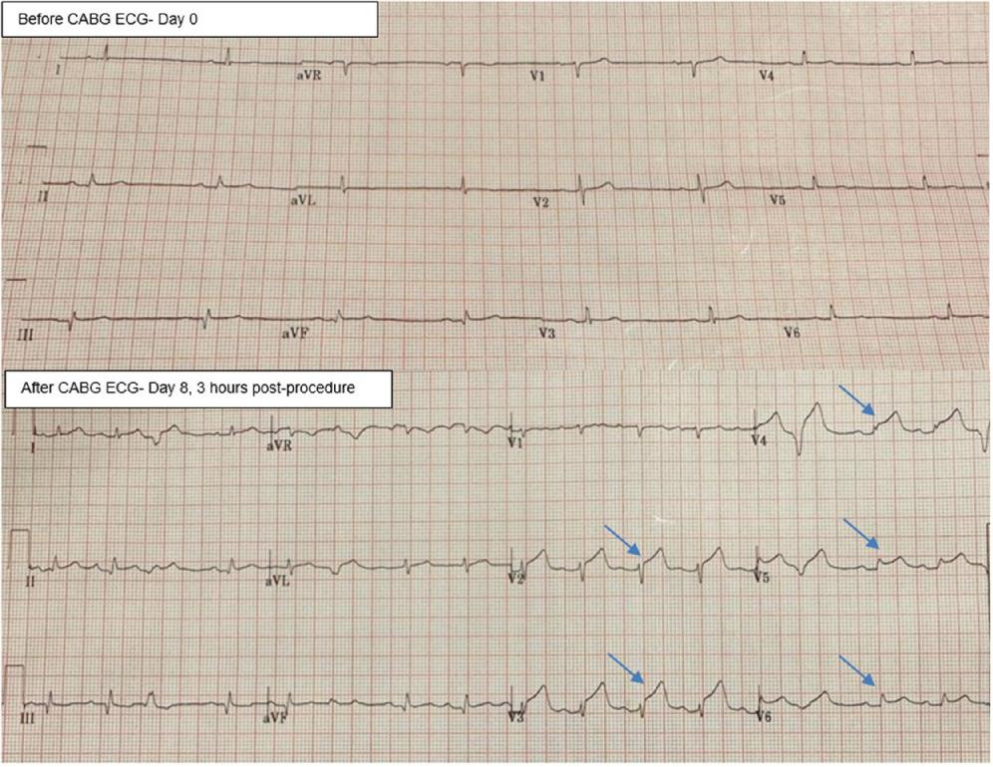
Before and after CABG ECGs showing persistent, new, ST elevation in leads V2–6 (arrows).

**Figure 2:**
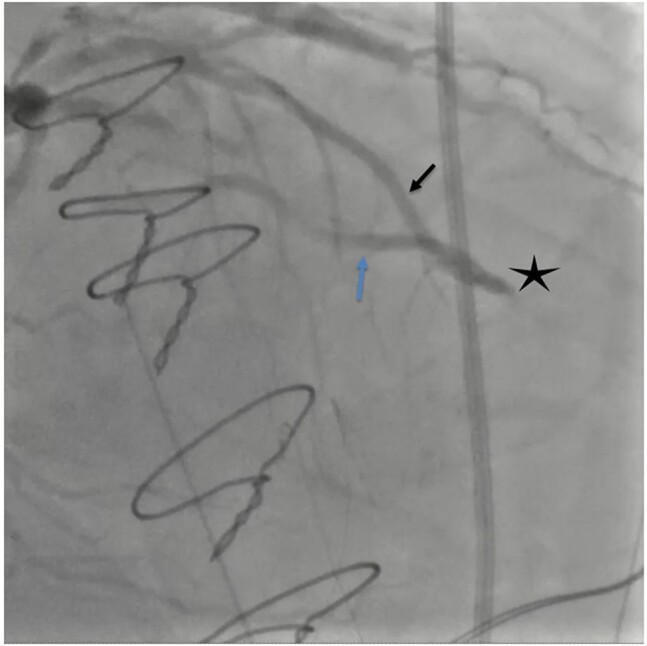
Post-CABG diagnostic angiography of the left coronary systemic via native vessels showing ante-grade filling of the left anterior descending artery (LAD, black arrow), occlusion of the mid-LAD at the site of the left internal mammary artery (LIMA) insertion (black star), and retrograde filling of the LIMA (blue arrow) (right anterior oblique view with cranial angulation).

**Figure 3:**
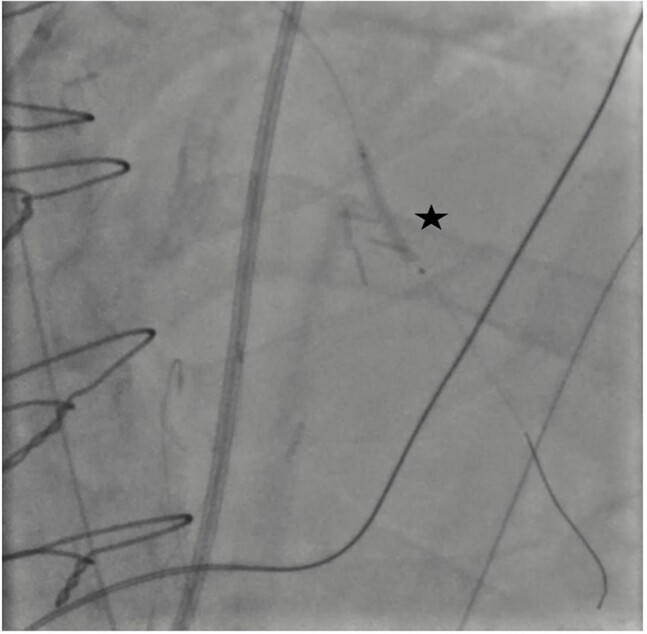
Postero-anterior view with cranial angulation showing drug-eluting balloon inflation (black star).

**Figure 4:**
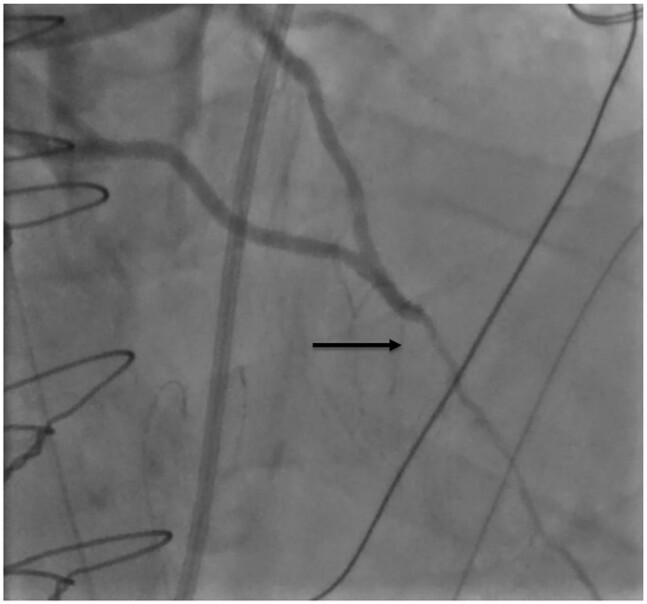
Right anterior oblique view with cranial angulation post-angioplasty angiogram showing re-established flow in the left anterior descending artery (black arrow).

A man in his 60 s was referred for ST elevation in anterior leads immediately after CABG surgery. He was an active smoker and known to have ischaemic heart disease and hyperlipidaemia and presented several days prior with chest pain and was diagnosed with non-ST-elevation myocardial infarction. He was on aspirin 75 mg once daily (o.d.), atorvastatin 80 mg o.d., ramipril 2.5 mg twice daily, and bisoprolol 2.5 mg o.d. Post-operatively, he received morphine intravenously and was on low-dose noradrenaline for blood pressure support. A post-operative 12-lead electrocardiogram (ECG) showed a new 2 mm ST elevation in leads V2, 3, and 4, and a 1 mm ST elevation in V5–6 (*Figure 1*). On examination, he had a sternotomy with a pericardial drain *in situ* and denied chest pain or shortness of breath. His blood pressure was 122/83 mmHg, heart rate of 89 beats/min, blood oxygen saturation of 96% on 2 L of inhaled oxygen, and no evidence of fluid overload. An echocardiogram was attempted, however, due to recent surgery, optimal echocardiographic windows could not be obtained. Due to dynamic ECG changes, further blood tests or radiological tests were deferred in favour of emergent coronary angiography, performed via the left radial artery (LRA). A 6 French (Fr) sheath was introduced and left ventriculography was performed with a 5 Fr Pigtail catheter, which showed new severe left anterior descending (LAD) artery territory hypokinesia. A 6 Fr Extra Back-up (EBU) 3.5 guiding catheter was used to engage the left coronary artery system and angiography was performed that confirmed a thrombotic occlusion of the mid-LAD at the site of the left internal mammary artery (LIMA) anastomosis, with contrast filling the latter retrogradely (*Figure 2* and Supplementary material online, *Video S1*) and unchanged disease elsewhere. Angiography of the venous grafts was performed with a 5 Fr Amplatz left 1 (AL1) diagnostic catheter that showed patent remaining grafts. Selective angiography of the free LIMA was not performed as the native vessel angiography already confirmed LAD occlusion with retrograde contrast filling of the LIMA. After discussion with the on-call cardiac surgeon, the freshly implanted free LIMA graft was considered at high risk of anastomotic suture tear with balloon angioplasty, therefore, the native vessels were engaged instead for coronary intervention. Intra-arterial unfractionated heparin (10 000 units) was administered and Sion (Vascular Perspectives, Holmfirth, UK) guidewire was used to negotiate the occlusion that was then pre-dilated with a 1.5 × 12 mm semi-compliant (SC) balloon at 18 atmospheres (ATM) with restoration of flow. A 2.25 × 20 mm paclitaxel-coated drug-eluting balloon (Medtronic, Watford, England) was then deployed at 12 ATM for 60 s (*Figure 3*), following which the LIMA graft started functioning again with evidence of competitive flow in distal LAD (see Supplementary material online, *Videos S2* and *S3* and *Figure 4*) and resolution of ST elevation. He continued standard treatment with aspirin 75 mg, once daily, and ticagrelor 90 mg, twice daily, for 12 months and lifelong aspirin thereafter. Since adequate arterial blood flow was established with no significant evidence of heavy clot burden, and with the additional risk of bleeding in the immediate post-operative phase, group IIb/IIIa inhibitors were not administered. He made an excellent recovery and was discharged home 10 days later. He remained stable cardiovascularly on outpatient follow-up at 5 months and continued to be free of angina. Of note, his left ventricular function completely normalized suggesting full myocardial recovery.

## Discussion

Coronary artery bypass graft remains an important mode of revascularization in patients with multi-vessel coronary artery disease.^4^ It represents a significant cardiovascular procedure and may be associated with peri-procedural myocardial infarction (MI) in 2% to 15% of the cases,^2^ with early graft occlusion as the cause in the majority of patients.^1^ This may occur due to endothelial injury that releases tissue factor, resulting in platelet activation and inflammation.^5^ Given our patient had a free LIMA graft due to intraoperative damage, the graft likely suffered a significant endothelial injury resulting in peri-operative thrombosis and occlusion. Other possibilities may include anastomotic imperfections, graft-native vessel mismatch, and pre-existing graft or native vessel pathology, especially at the site of anatamosis.^5^ Diagnosis may be challenging as patients are usually either intubated or administered opioid analgesics, blunting the common ischaemic symptoms such as chest pain.^2^ Cardiac enzymes may be elevated secondary to the procedure and can be difficult to interpret.^1,2^ ST-segment changes may also develop due to pericardial inflammation and are usually benign if occur after 24 h of the procedure.^2,6^ However, persistent, regional ST elevation within 24 h of the surgery is abnormal and shouldn’t be ignored as it may be suggestive of STEMI. One reason for this may be the early closure of grafts, reported to be as high as 3%.^2^ Sadana *et al.*^3^ describe a similar case where a patient underwent invasive angiography showing compromised flow in the LAD artery. An elevation in troponin to greater than 10 times the upper value, new wall motion abnormalities on cardiovascular imaging, or new development of Q waves further aids the diagnosis of MI.^7^ However, transthoracic echocardiography may be limited by suboptimal views. We faced similar challenges as our patient was administered significant doses of opiate analgesics, had no chest pain, and non-diagnostic transthoracic echocardiography. Since the ECG changes were persistent, we opted for emergent angiography that confirmed occlusion in the mid-LAD, at the site of the LIMA graft anastomosis that was likely adjacent to pre-existing atheroma, thereby disrupting it and leading to thrombosis. An incorrectly positioned anastomotic suture of the back wall of the LAD was also speculated however, since the wire and balloon crossed easily, we felt this was unlikely in our case.

Management of early post-CABG MI may be challenging. Drug-eluting stents may be used to re-establish coronary flow in cases where the STEMI is caused due to graft or native vessel occlusion.^1,3,7^ However, deployment across the anastomosis (bifurcation) would rule out any future intervention for chronically occluded coronary arteries. This may be further complicated by graft dehiscence, perforation, or embolization, with the risk rising with sequential balloon inflations that may often be required to achieve optimal stent results.^1^ Drug-eluting balloons may represent a safer alternative.^8^ Studies have shown good results in the context of in-stent restenosis when treated with DEBs.^8,9^ Benefits include the absence of metal implantation, thereby, allowing early cessation of dual-antiplatelet agents, if required.^8,9^ Similarly, since neither the graft nor the native vessel is jailed behind stent struts, DEBs preserve access for future coronary intervention. Hybrid CABG-DEB procedures, whereby graft-assisted distal vessel DEB angioplasty is performed intraoperatively, have been described.^10^ However, such procedures were planned prior to surgery and not in the context of post-CABG STEMI management.^10^ Moreover, the arterial access sheath was inserted into the non-anastomotic end of the graft under direct vision intraoperatively and not percutaneously via a peripheral access.^10^ Our patient developed STEMI in the immediate post-operative stage. Using left radial access, we approached the occlusion via the native coronary vessels as the free LIMA graft was deemed at high risk of complications and successfully re-established blood flow with a semi-compliant angioplasty balloon followed by a DEB, resulting in re-functioning of the LIMA and resolution of ST-segments. Since the LIMA started to function again, we didn’t treat the severe proximal LAD calcified stenosis. One option would have been to stop after plain old balloon angioplasty with a semi-compliant balloon, however, this may be associated with higher restenosis due to vessel recoil from associated injury in comparison to a DEB and therefore, the latter was deployed as well.^9^ The patient made an excellent recovery and was discharged 10 days later, remaining stable cardiovascularly at the 5-month follow-up review. To our knowledge, this case represents the first instance worldwide of a DEB used for STEMI immediately after CABG surgery with satisfactory results and no immediate complications.

## Supplementary Material

ytae245_Supplementary_Data

## Data Availability

The data underlying this article are available in the article and in its online Supplementary material.

## Lead author biography

Dr Muhammad Usman Shah is a higher speciality interventional cardiology trainee and a cardiometabolic research fellow and PhD candidate at the University of Lincoln. His interests lie in complex coronary intervention and cardiometabolic medicine.

## Supplementary material

**Consent:** The authors confirm that written consent for submission and publication of this case including images and associated text has been obtained from the patient in line with COPE guidance.

**Funding:** None.
